# Redox Controls on Dissolved Metal Distribution and Screening-Level Health Risks in Groundwater of the Chiang Mai Basin, Northern Thailand

**DOI:** 10.3390/toxics14050390

**Published:** 2026-04-30

**Authors:** Rungroj Benjakul, Sutthipong Taweelarp, Morrakot Khebchareon, Schradh Saenton, Nipada Santha

**Affiliations:** 1 Department of Geological Sciences, Faculty of Science, Chiang Mai University, Chiang Mai 50200, Thailand; rungroj.b@cmu.ac.th; 2 Department of Geotechnology, Faculty of Technology, Khon Kaen University, Khon Kaen 40002, Thailand; sutthita@kku.ac.th; 3 Department of Mathematics, Faculty of Science, Chiang Mai University, Chiang Mai 50200, Thailand; morrakot.k@cmu.ac.th; 4 Environmental Science Research Center, Faculty of Science, Chiang Mai University, Chiang Mai 50200, Thailand

**Keywords:** groundwater quality screening, hydrochemical evolution, redox-sensitive metal mobilization, well-depth stratification, drinking-water health risk

## Abstract

Groundwater contamination by dissolved metals and metalloids in the Chiang Mai Basin is an important drinking-water concern, yet the coupled depth patterns, hydrogeochemical controls, composite contamination status, and screening-level health implications have not previously been assessed in an integrated basin-scale framework. This study evaluated 120 groundwater samples from alluvial wells classified by depth as shallow (≤30 m, *n* = 40), intermediate (31–60 m, *n* = 35), and deep (>60 m, *n* = 45). Samples were analyzed for nine dissolved metals and metalloids (Fe, Mn, As, Cd, Pb, Cr, Zn, Hg, and Se) together with pH, Eh, and total dissolved solids (TDS). The highest exceedance frequencies were observed for Fe (72.5% of samples, >0.3 mg/L acceptability threshold), Mn (65.0%, >0.08 mg/L), and As (45.8%, >10 μg/L). Fe and Mn increased significantly with depth, whereas As was enriched in deep wells but showed no statistically significant depth dependence. Pearson correlation and principal component analysis consistently identified a dominant redox-associated component in which Fe, Mn, and As covaried negatively with Eh, supporting redox-sensitive co-enrichment in deeper groundwater. Contamination factors calculated relative to selected global groundwater background values were >6 for all seven evaluated metals (Fe, Mn, As, Cd, Pb, Cr, and Zn), and the overall pollution load index (PLI) was 9.11, with the highest depth-specific PLI in deep wells (10.42). These indices are interpreted here as background-relative screening tools rather than stand-alone regulatory measures. A screening-level ingestion risk assessment identified arsenic as the dominant toxicological driver, with hazard quotients (HQ) of 1.97 for adults and 4.60 for children, and an estimated lifetime cancer risk (LCR) of 8.87 × 10^–4^. The results support targeted monitoring of deeper wells, routine screening for As and Mn, and treatment strategies that can address the co-occurring Fe–Mn–As assemblage in alluvial groundwater.

## 1. Introduction

Groundwater is a critical resource for drinking water supply, agriculture, and drought resilience in rapidly urbanizing regions worldwide [1,2]. Chiang Mai in northern Thailand is experiencing increasing population growth, tourism, peri-urban expansion, and agricultural demand [3]. These pressures place increasing stress on surface-water systems in the area [4,5]. Consequently, groundwater has become an important drinking-water source in the principal intermontane basin of the Chiang Mai Basin.

The combination of complex intermontane hydrogeology and growing pressure from urbanization in the Chiang Mai Basin has contributed to a gradual decline in groundwater quality for drinking-water use [4,5]. Previous work in the Chiang Mai Basin reported locally high iron and manganese concentrations in groundwater [6]. In the neighboring Lampang Basin, northern Thailand, elevated arsenic concentrations have been linked to carcinogenic risk [7]. More broadly, intermontane basins in northern Thailand commonly contain elevated Fe, Mn, and As, especially where groundwater evolves toward more reducing conditions with depth [6,7]. However, an integrated basin-scale assessment linking depth distribution, hydrogeochemical behavior, composite contamination indices, and health-risk screening has been lacking for the Chiang Mai Basin itself.

In alluvial groundwater systems, dissolved-metal occurrence is controlled not only by aquifer lithology but also by geochemical transformation along flow paths. Redox evolution is particularly important because decreasing oxygen availability and lower Eh can destabilize Mn(IV)- and Fe(III)-bearing oxide phases, releasing Fe, Mn, and sorbed or co-precipitated trace elements such as arsenic to groundwater [8,9,10]. Similar multi-metal patterns have been documented across South and Southeast Asia, making the Chiang Mai Basin an important case study for regional comparison [1,2,11].

Dissolved metals and metalloids in groundwater can be highly toxic even at low exposure levels, particularly arsenic, manganese, cadmium, and lead [12]. Arsenic remains the principal toxicological concern in many reducing alluvial aquifers because chronic ingestion has been associated with cancer and multiple systemic effects [13,14,15]. Manganese, cadmium, and lead are also relevant from a drinking-water perspective, although their hydrogeochemical behavior and toxicological assessment frameworks differ [16]. In addition to comparing measured concentrations with drinking-water thresholds, composite indices such as the contamination factor (CF) and pollution load index (PLI) can be useful as background-relative screening tools for comparing deviations from selected baseline concentrations across multiple metals [17,18,19,20].

Recent work has reinforced that naturally elevated arsenic in alluvial aquifers is rarely controlled by a single factor. Instead, sediment source, depositional architecture, groundwater residence time, organic matter availability, and pumping-driven hydrologic change can interact to create spatially heterogeneous risk fields even within the same basin [21,22,23]. This broader understanding is important for the Chiang Mai Basin because it suggests that simple concentration mapping alone is insufficient; geochemical interpretation and depth-sensitive risk screening are also needed to identify which wells are most likely to deteriorate as redox conditions evolve [24,25].

In addition, manganese has emerged as an increasingly important co-contaminant in Asian groundwater systems. Recent perspective papers have argued that manganese deserves more systematic attention because it frequently co-occurs with arsenic and iron [26,27,28], may remain overlooked in routine domestic-well monitoring, and has distinct health relevance for infants and children [29,30]. The Chiang Mai Basin dataset therefore offers value not only as a local groundwater-quality assessment, but also as a contribution to the wider regional discussion on coupled arsenic–manganese groundwater risk in Asia [22,27,28,31].

Accordingly, the objectives of this study were to: (1) characterize the vertical distribution of dissolved metals and metalloids in shallow, intermediate, and deep groundwater; (2) evaluate redox-sensitive relationships among metals and hydrochemical variables using correlation analysis and PCA; (3) assess composite contamination using CF and PLI relative to selected background values; and (4) estimate screening-level non-carcinogenic and carcinogenic risks associated with groundwater ingestion, with emphasis on arsenic as the principal carcinogenic constituent in the dataset.

## 2. Materials and Methods

### 2.1. Study Area

**Figure 1:**
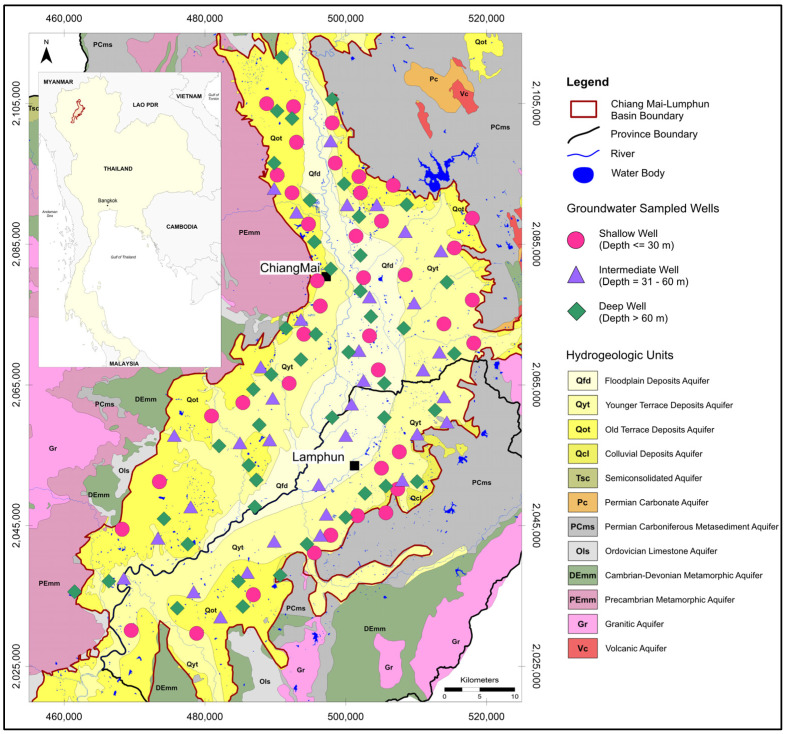
Maps of the Chiang Mai Basin hydrogeologic units and sampled well locations.

The Chiang Mai groundwater basin is an intermontane alluvial basin in northern Thailand (see Figure 1). The basin is underlain by Quaternary unconsolidated sediments and is widely used for domestic, agricultural, and municipal water supply. Hydrostratigraphically, the system includes floodplain, younger terrace, and older terrace deposits, with groundwater abstraction from both relatively shallow and deeper wells across the basin [4,5].

From a hydrogeological perspective, this basin setting is conducive to vertically variable groundwater chemistry. Basin-margin recharge, fine-grained central floodplain deposits, and differences in groundwater circulation length can create local zones where oxidizing and reducing waters coexist over relatively short distances. Such heterogeneity helps explain why dissolved trace-metal occurrence in alluvial aquifers is often patchy rather than uniform and why depth-stratified interpretation is particularly important for Chiang Mai Basin groundwater management.

### 2.2. Groundwater Sampling and Field Measurements

A total of 120 groundwater samples from monitoring and domestic wells were evaluated in this study. Based on the hydrogeological characteristics in Chiang Mai Basin, aquifer can be characterized largely as an unconfined aquifer, extends to an average depth of 30 m, while the deeper than 30 m as confined aquifers [4,5]. Moreover, groundwater development patterns within the Chiang Mai Basin mainly reveals three distinct depths including ≤30 m, 31–60 m, and >60 m [3]. Therefore, the sampling wells were classified into three depth categories: shallow (depth ≤ 30 m, *n* = 40), intermediate (depth = 31–60 m, *n* = 35), and deep (depth > 60 m, *n* = 45). Prior to sampling, wells were purged until field parameters (pH, redox potential (Eh), temperature, and electrical conductivity (EC) stabilized to obtain representative groundwater. These parameters were measured in situ using a calibrated portable multi-parameter meter (Hach HQ40d) with automatic temperature compensation, and total dissolved solids (TDS) were calculated automatically from EC. Subsamples for dissolved-metal analysis were filtered through 0.45 μm cellulose acetate membranes and collected in pre-cleaned 200 mL HDPE bottles. Samples were immediately acidified to pH < 2 with ultrapure HNO_3_ (65%, Merck^®^, Darmstadt, Germany). All samples were labeled, tightly sealed, and transported in chilled containers to the laboratory for storage at 4 °C. The dataset used here represents a basin-wide screening dataset rather than a seasonal monitoring series.

This depth grouping was selected to balance hydrostratigraphic interpretability and sample size. It also produces a practical management classification that is meaningful for domestic and community wells because well depth is usually known even when screened intervals and detailed construction records are unavailable. Throughout the manuscript, depth is therefore used as a surrogate for hydrogeochemical position within the basin rather than as a stand-alone causal variable.

### 2.3. Laboratory Analysis

Nine dissolved metals and metalloids (Fe, Mn, Zn, Cd, Hg, Cr, As, Se, and Pb) were analyzed using inductively coupled plasma mass spectrometry (ICP-MS; Agilent 7900, Santa Clara, CA, USA). Quality assurance/quality control included calibration-verification checks every 5 samples and procedural blanks every 5 samples. Moreover, each analyte also was measured triplicate per sample. Because the present screening dataset does not include a complete major-ion dataset for all samples, charge-balance evaluation was not used as a primary QA/QC criterion for the dissolved trace-metal dataset. Hg and Se were retained in the descriptive and compliance assessment because they were included in the analytical program, but they were not emphasized in the multivariate interpretation because concentrations were low and no sample exceeded the relevant WHO guideline values in the present dataset.

Because redox-sensitive elements can be affected by field handling and preservation, the analytical results are interpreted as dissolved concentrations at the time of sampling rather than as complete operational speciation. The present dataset is therefore suitable for evaluating co-occurrence patterns, guideline exceedance, and screening-level risk, but it cannot independently distinguish dissolved ferrous (Fe^II^) from ferric (Fe^III^) iron or arsenite (As^III^) from arsenate (As^V^). This limitation is important when discussing mechanisms and is explicitly considered in the interpretation sections below.

### 2.4. Statistical Analysis

Statistical analysis was performed to characterize the distribution of hydrochemical parameters and dissolved metals and metalloids, evaluate groundwater-quality exceedances, and examine both inter-variable relationships and vertical variation with depth. Descriptive statistics were calculated for pH, Eh, TDS, Fe, Mn, Zn, Cd, Hg, Cr, As, Se, and Pb for the full dataset and for three depth classes: shallow (0–30 m), intermediate (31–60 m), and deep (>60 m). For each parameter, values are reported as mean ± SD together with the minimum and maximum. The frequency of wells exceeding the WHO reference values used in this study was also determined. These reference values were applied as screening criteria for groundwater-quality evaluation; some parameters, including Fe and TDS, represent operational or aesthetic benchmarks rather than strictly health-based limits. Pearson correlation coefficients were calculated among selected metals (Fe, Mn, As, Cd, Pb, Cr, and Zn) and hydrochemical variables (pH, Eh, and TDS) to assess possible geochemical associations and co-mobilization patterns. Because the data were not assumed to be normally distributed, differences among the three depth groups were tested using the Kruskal–Wallis test [32]. This analysis was used to determine whether the distributions of hydrochemical parameters and dissolved metals and metalloids differed significantly among shallow, intermediate, and deep groundwater. A *p*-value < 0.05 was considered statistically significant for the Kruskal–Wallis test, whereas correlations were considered significant at *p* < 0.01.

Principal component analysis (PCA) was performed on z-score-standardized variables (Fe, Mn, As, Cd, Pb, Cr, Zn, pH, Eh, and TDS). Sampling adequacy was acceptable for exploratory PCA (KMO = 0.69), and Bartlett’s test of sphericity was significant (*p* < 0.05), confirming matrix factorability. Three interpretable components were retained on the basis of the scree pattern and loading structure, and varimax rotation was used to aid interpretation. Specifically, component retention was guided by the Kaiser criterion (eigenvalue > 1.0), which identified three components with eigenvalues of 2.57, 1.34, and 1.13, respectively; the fourth component had an eigenvalue of 0.98 and was not retained. The scree plot confirmed a clear inflection after the third component. Because only three of ten components were retained, the cumulative explained variance was 50.4%, which is moderate but consistent with what is typically observed in exploratory PCA of heterogeneous environmental datasets where multiple overlapping natural and anthropogenic processes contribute to the covariance structure [32]. The PCA is therefore treated as an exploratory pattern-recognition tool rather than an exhaustive variance decomposition, and its results are interpreted in conjunction with the Pearson correlation analysis and depth-stratified patterns presented in Section 3.4 and Section 3.5. The statistical framework was designed primarily for pattern recognition rather than formal source apportionment. In hydrochemical datasets of this type, skewness and outliers are common because a relatively small number of wells may intersect localized reducing zones or enriched sedimentary facies. For that reason, the manuscript reports mean together with medians and range statistics, and the multivariate tools are interpreted as exploratory lines of evidence that help organize the observed covariance structure rather than prove a unique mechanism.

### 2.5. Contamination Assessment Indices

Composite contamination status was screened using the contamination factor (CF) and pollution load index (PLI). CF for each metal was calculated using Equation (1) [17,33].

(1) $${\mathrm{CF}}_{i}={\bar{C}}_{i}/C_{i}^{b},$$

where ${\bar{C}}_{i}$ is the mean measured concentration of metal *i* and $C_{i}^{b}$ is the selected background concentration based on reported global natural groundwater reference values (Fe = 0.200 mg/L, Mn = 0.050 mg/L, As = 1.0 μg/L, Cd = 0.1 μg/L, Pb = 1.0 μg/L, Cr = 1.0 μg/L, and Zn = 10 μg/L) from Reimann and de Caritat [34]. The contamination factor was interpreted using four classes: CF < 1, low contamination; 1 ≤ CF < 3, moderate contamination; 3 ≤ CF < 6, considerable contamination; and CF ≥ 6, very high contamination [35].

PLI was calculated as the *n*th root of the product of the individual CF values across the seven evaluated metals, as shown in Equation (2). The resulting PLI was interpreted as an index of overall site quality. PLI < 1 indicates low pollution, PLI = 1 indicates baseline conditions, and PLI > 1 indicates progressive site deterioration [36].

(2) $$\mathrm{PLI}={\left[\prod_{i=1}^{n}{\mathrm{CF}}_{i}\right]}^{1/n}={\left[{\mathrm{CF}}_{\mathrm{Fe}}\times {\mathrm{CF}}_{\mathrm{Mn}}\times {\mathrm{CF}}_{\mathrm{As}}\times {\mathrm{CF}}_{\mathrm{Cd}}\times {\mathrm{CF}}_{\mathrm{Pb}}\times {\mathrm{CF}}_{\mathrm{Cr}}\times {\mathrm{CF}}_{\mathrm{Zn}}\right]}^{1/7}$$

In this study, CF and PLI are interpreted as background-relative screening metrics and not as direct regulatory surrogates. These indices were retained in the revised manuscript because they provide a compact way to summarize departures from baseline concentrations across multiple constituents. However, they were used explicitly as screening metrics rather than regulatory indicators. This distinction is especially important in groundwater studies because CF and PLI are highly sensitive to the selected background values and may appear elevated even where drinking-water risk is driven mainly by only one or two constituents.

### 2.6. Screening-Level Human Health Risk Assessment

Human health risk was assessed for groundwater ingestion as a screening-level exposure pathway following the USEPA framework [37]. Risk was divided into non-carcinogenic and carcinogenic effects depending on the toxicity of each metal. Non-carcinogenic effects were quantified as hazard quotients (HQ), Equation (3), and the cumulative hazard index (HI) was obtained by summing HQ values, Equation (4), for As, Mn, Cd, and Zn only. Iron was excluded from the cumulative HQ because it is primarily an acceptability-related constituent in drinking water and is unlikely to cause adverse health effects at the concentrations observed over a lifetime of ingestion. Lead was excluded because EPA IRIS does not provide a standard oral RfD suitable for routine HQ derivation for inorganic lead [38]. Total chromium was also excluded from cancer calculations because chromium speciation (Cr^III^/Cr^VI^) was not determined. Accordingly, Cr-related health risk is interpreted cautiously as a screening estimate based on total dissolved chromium rather than a speciated toxicological assessment. It should be noted that cadmium was retained in the non-carcinogenic HQ calculation but excluded from the carcinogenic risk assessment because approximately 90% of samples had concentrations below the WHO guideline of 0.003 mg/L (Table 1), and was therefore screened out as a negligible contributor to cumulative carcinogenic risk following standard risk assessment protocols.

(3) $$\mathrm{HQ}=\frac{\mathrm{ADD}}{\mathrm{RfD}}$$

(4) $$\mathrm{HI}={\mathrm{HQ}}_{\mathrm{As}}+{\mathrm{HQ}}_{\mathrm{Mn}}+{\mathrm{HQ}}_{\mathrm{Cd}}+{\mathrm{HQ}}_{\mathrm{Zn}}$$

ADD is the average daily dose (mg/kg/day), calculated using Equation (5)

(5) $$\mathrm{ADD}\;=\frac{C\times \;\mathrm{IR}\;\times \;\mathrm{EF}\;\times \;\mathrm{ED}}{\mathrm{BW}\;\times \;\mathrm{AT}},$$

where C is the concentration of each metal in groundwater (mg/L), IR is the ingestion rate (2.0 L/day for adults and 1.0 L/day for children), EF is the exposure frequency (365 days/year), ED is the exposure duration (30 years for adults and 6 years for children for non-carcinogenic risk) [39], BW is body weight (70 kg for adults and 15 kg for children), and AT is the averaging time (10,950 days for adults and 2190 days for children for non-carcinogenic risk) [39]. Under these assumptions, the equation simplifies to ADD = C × IR/BW [37,40]. Oral reference doses used were As = 3.0 × 10^–4^ [41,42], Mn = 1.4 × 10^–1^ [43], Cd = 5.0 × 10^–4^ [44], and Zn = 3.0 × 10^–1^ [45,46] mg/kg/day, consistent with values commonly used in groundwater health-risk studies.

Lifetime cancer risk was estimated only for inorganic heavy metals and metalloids that were higher than regulatory safety thresholds (WHO) using Equation (6).

(6) CR = LADD × CSF,

where LADD is the lifetime average daily dose, calculated for an adult resident lifetime screening scenario using the same basic approach as ADD and assuming a 70-year lifespan, Equation (3). CSF is the oral slope factor for inorganic arsenic, taken as 1.5 (mg/kg-day)^−1^. Consistent with EPA screening practice, cancer risk values above 1 × 10^–4^ were interpreted as elevated screening-level concern [37,47]. In addition to mean-concentration risk estimates, the 90th-percentile arsenic concentration was evaluated to illustrate higher-risk wells within the dataset.

This assessment is intended as a screening-level rather than site-specific risk characterization. Several exposure variables, including household treatment practices, actual water consumption, alternative water sources, and temporal variability in well chemistry, were not available in the raw dataset. The risk estimates are therefore most appropriately interpreted as prioritization tools that identify which contaminants and well groups warrant closer monitoring and, where necessary, more detailed follow-up assessment using household- or community-specific exposure information.

## 3. Results

### 3.1. Physicochemical Conditions and Drinking-Water Compliance

Groundwater pH ranged from 6.0 to 8.8, with a mean of 7.21 and a median of 7.18 (Table 1). Only 5.8% of samples fell outside the recommended pH range of 6.5–8.5, whereas Eh ranged from −120.7 to +327.7 mV (mean +125.3 mV), indicating conditions from moderately oxidizing to reducing groundwater. Shallow and intermediate wells were predominantly oxidizing, whereas deep groundwater tended to be more reducing. TDS ranged from 231.8 to 1111.5 mg/L (mean 484.6 mg/L), and 40.0% of samples exceeded the 500 mg/L acceptability threshold.

Analysis of the nine dissolved metals and metalloids (Fe, Mn, Zn, Cd, Hg, Cr, As, Se, and Pb) showed that Fe exceeded the 0.3 mg/L acceptability threshold in 72.5% of all samples, followed by Mn and As. Using the current WHO provisional guideline values adopted in this study, Mn exceeded the 0.08 mg/L threshold in 65.0% of samples, whereas As exceeded 10 μg/L in 45.8% of samples. Cd and Pb exceeded their guideline values of 3 μg/L and 10 μg/L in 11.7% and 20.0% of samples, respectively, whereas no sample exceeded the guideline values for Hg, Cr, or Se.

The exceedance pattern indicates that groundwater-quality deterioration in the basin is not controlled by a single constituent alone. Instead, the basin shows a mixed portfolio of health-based and acceptability-based concerns: arsenic and manganese drive the main human-health concern, whereas iron and TDS affect potability, appearance, and treatment demand. Framing the results in this way helps distinguish parameters that primarily indicate redox evolution from those that directly motivate public-health intervention.

### 3.2. Dissolved Metal and Metalloid Concentrations and Vertical Distribution

#### 3.2.1. Spatial Patterns of Dissolved Metal and Metalloid Concentrations

**Figure 2:**
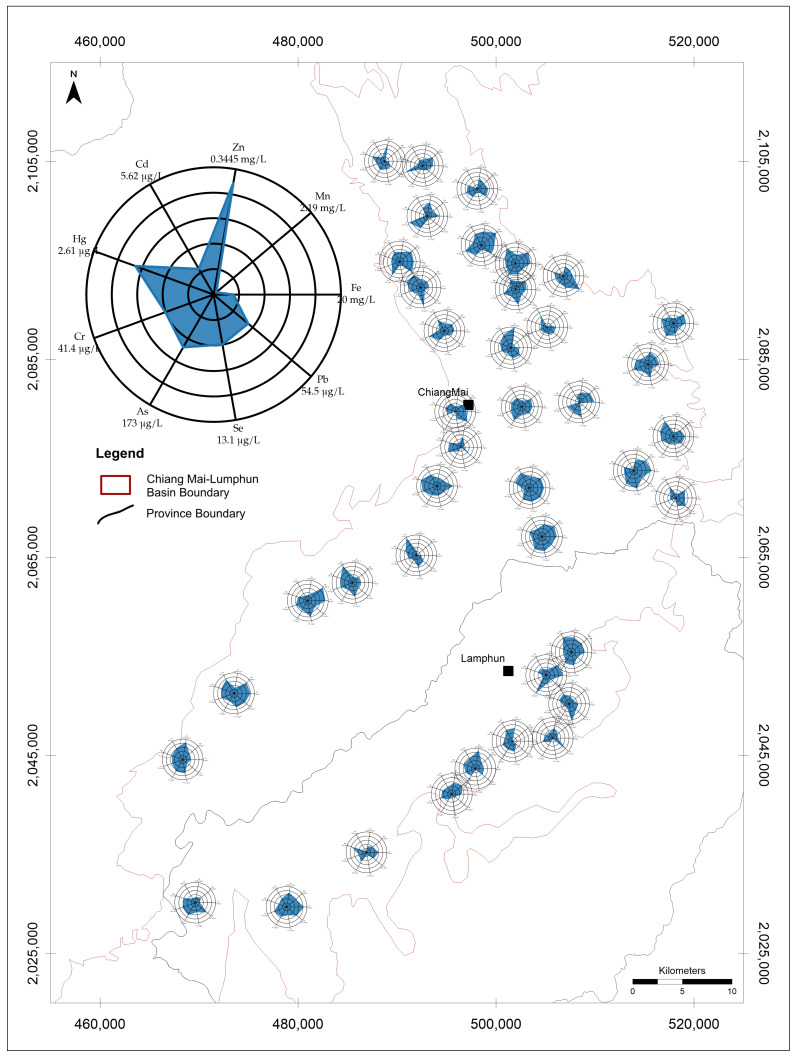
Dissolved metal(loid) distribution in shallow groundwater (0–30 m) of the Chiang Mai Basin.

**Figure 3:**
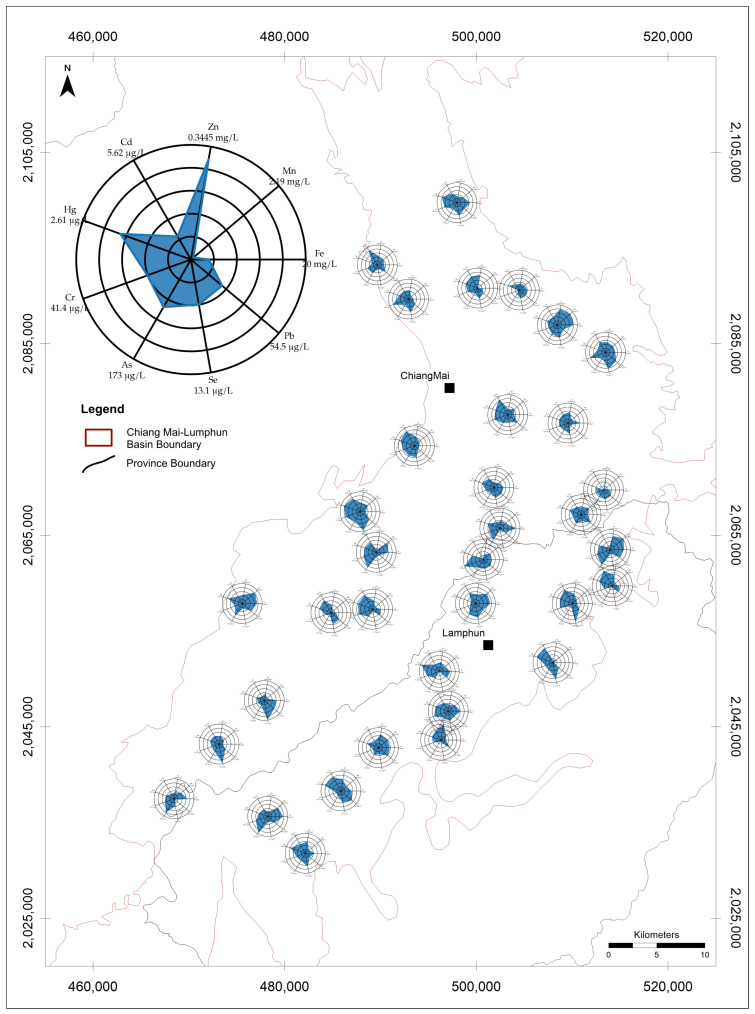
Dissolved metal(loid) distribution in intermediate groundwater (31–60 m) of the Chiang Mai Basin.

**Figure 4:**
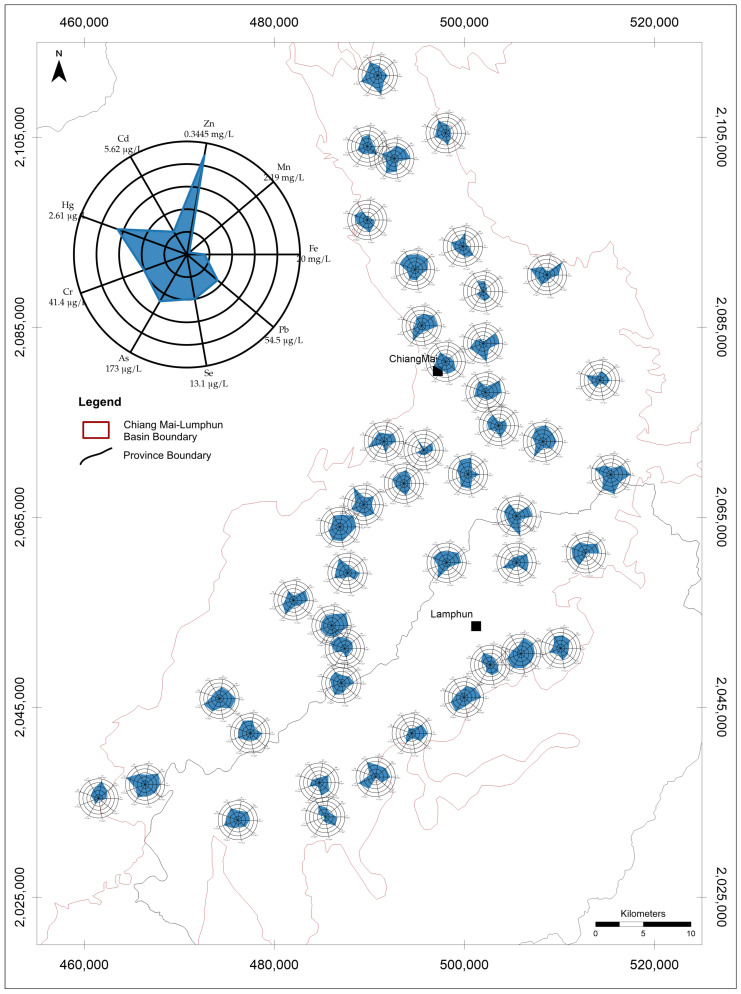
Dissolved metal(loid) distribution in deep groundwater (>60 m) of the Chiang Mai Basin.

The results of all metal and metalloids contents have shown as Table S1. Dissolved metal(loid) concentration maps combined with radar diagrams for shallow, intermediate, and deep groundwater revealed pronounced spatial heterogeneity across the study area (Figure 2, Figure 3 and Figure 4). The radar polygons varied substantially among wells, indicating that dissolved metal(loid) enrichment was localized rather than uniformly distributed throughout the basin. This spatial variability suggests that dissolved metal occurrence was controlled by local hydrogeochemical conditions rather than by a single basin-wide contamination process.

The radar diagrams should be interpreted with caution because each axis was scaled to the maximum concentration observed for that individual metal. Accordingly, polygon geometry reflects the relative enrichment of each element within a given well but does not represent a direct comparison of absolute concentrations among different metals. Thus, although Zn, As, Cd, Se, or Pb may appear visually prominent in some radar plots, the raw concentration data indicate that Fe and Mn dominated the dissolved metal load in the groundwater system (Table 1).

Across the full dataset, Fe had the highest overall mean concentration (1.856 mg/L), followed by Mn (0.311 mg/L) and Zn (66.5 μg/L) (Table 1). Arsenic showed a lower mean concentration (20.7 μg/L) but strong positive skewness, with a median of 8.9 μg/L and a maximum of 173 μg/L. By contrast, Cd occurred at much lower concentrations overall, with a maximum of 5.6 μg/L. These results indicate that Fe and Mn were the dominant dissolved metals in absolute terms, whereas As was characterized by lower average concentration but occasional high-concentration occurrences.

#### 3.2.2. Vertical Distribution Among Shallow, Intermediate, and Deep Wells

**Figure 5:**
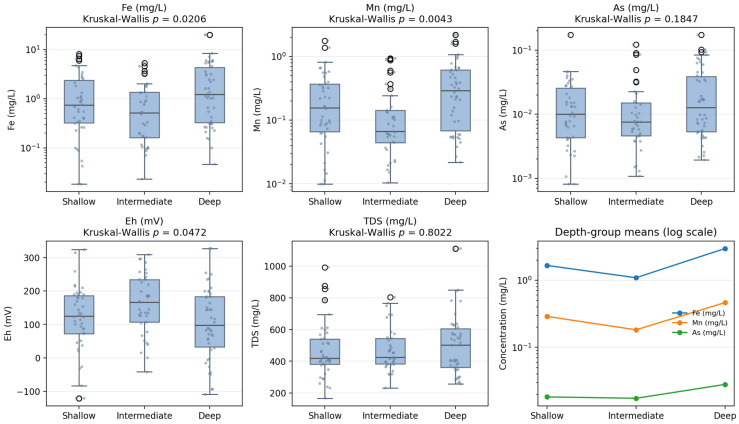
Box plots of Fe, Mn, and As concentrations (plotted on a base-10 logarithmic y-axis) and Eh and TDS values for various groundwater depth groups: shallow (0–30 m), intermediate (31–60 m), and deep (>60 m), with Kruskal–Wallis test results shown. Kruskal–Wallis tests revealed significant depth-related differences for Fe, Mn, and Eh, but not for As or TDS. Line plot comparing depth-group mean concentrations of Fe, Mn, and As on a log_10_ scale.

Comparison of concentration data among the three depth intervals showed that the clearest vertical pattern was associated with Fe and Mn enrichment in deep groundwater. Mean Fe concentrations were 1.684 mg/L in shallow wells, 1.077 mg/L in intermediate wells, and 2.614 mg/L in deep wells, while mean Mn concentrations were 0.293, 0.180, and 0.427 mg/L, respectively (Table 1). The corresponding box plots also illustrate the upward shift in Fe and Mn distributions in the deep wells relative to the other depth classes (Figure 5).

Kruskal–Wallis testing confirmed significant differences among depth groups for Fe (H = 7.60, *p* = 0.0224) and Mn (H = 10.75, *p* = 0.0046) (Table 2). Notably, the intermediate-depth wells showed the lowest mean concentrations for both metals, indicating that the vertical pattern was not strictly monotonic but instead followed a shallow–intermediate–deep structure in which the deepest interval was most enriched. Arsenic showed a weaker vertical trend than Fe and Mn. Mean As concentrations were 18.6 μg/L in shallow wells, 17.7 μg/L in intermediate wells, and 25.0 μg/L in deep wells (Table 1). Although the highest mean value occurred in the deep aquifer, the Kruskal–Wallis result was not statistically significant (H = 3.15, *p* = 0.2067). The box plots likewise show substantial overlap in As concentrations among the three depth groups (Figure 5). Therefore, while As tended to be higher in deep groundwater on average, the present dataset does not support a statistically robust depth effect.

The remaining trace metals showed either weak or inconsistent vertical variation (Table 2; Figure 5). Zn concentrations were similar among the three depth classes, with means of 70.6 μg/L, 57.8 μg/L, and 69.8 μg/L in shallow, intermediate, and deep wells, respectively. Pb was slightly higher in shallow wells, whereas Hg and Se were marginally higher in intermediate wells. However, none of these variations were statistically significant. Overall, the results indicate that vertical differentiation in groundwater metal chemistry was most pronounced for Fe and Mn, whereas other metals and metalloids exhibited relatively limited depth dependence.

#### 3.2.3. Hydrochemical Indicators Related to Vertical Distribution

The vertical distribution of heavy metals was accompanied by a significant depth-related difference in redox conditions. Eh differed significantly among the three well-depth groups (H = 7.09, *p* = 0.0289), with the lowest mean Eh observed in deep groundwater (Table 2). In contrast, TDS showed no significant variation with depth (H = 0.029, *p* = 0.9854), and the distributions overlapped extensively among the three groups (Figure 5).

This contrast between Eh and TDS indicates that the strongest vertical hydrochemical signal was not simply an increase in total dissolved solids with depth. Instead, the combination of lower Eh and higher Fe-Mn concentrations in deep wells suggests that vertical changes in groundwater chemistry were linked more closely to redox evolution than to generalized solute accumulation. In this dataset, Fe and Mn therefore represent the most robust indicators of depth-related hydrochemical change.

### 3.3. Contamination Factors and Pollution Load Index

Using the selected global groundwater background values of Reimann and de Caritat [30], all seven evaluated metals and metalloids had CF values greater than 6.0, corresponding to the very high contamination class within the index framework (Table 3). Mean CF values were highest for As (20.71), followed by Cd (13.65), Fe (9.28), Cr (7.20), Pb (6.68), Zn (6.65), and Mn (6.21). The overall PLI for the 120-well dataset was 9.11, indicating progressive contamination relative to the selected baseline values.

Depth-specific PLI values were 8.93 for shallow wells, 7.29 for intermediate wells, and 10.42 for deep wells, indicating the greatest composite background-relative deviation in the deepest groundwater group. Because these metrics depend strongly on the selected baseline concentrations, they are interpreted here as comparative screening indices rather than direct evidence of regulatory non-compliance or direct proof of severe basin deterioration in a public-health sense.

At the same time, the moderate difference between shallow and intermediate wells compared with the stronger separation of the deep group suggests that the composite index is responding to a basin-scale geochemical gradient rather than random noise alone. Even so, the depth-specific PLI pattern should not be interpreted as proof that all deep wells are uniformly contaminated. Instead, it indicates that the deepest group contains the strongest overall departure from the selected global baseline and therefore deserves closer spatial and well-specific inspection.

### 3.4. Correlation Analysis

**Figure 6:**
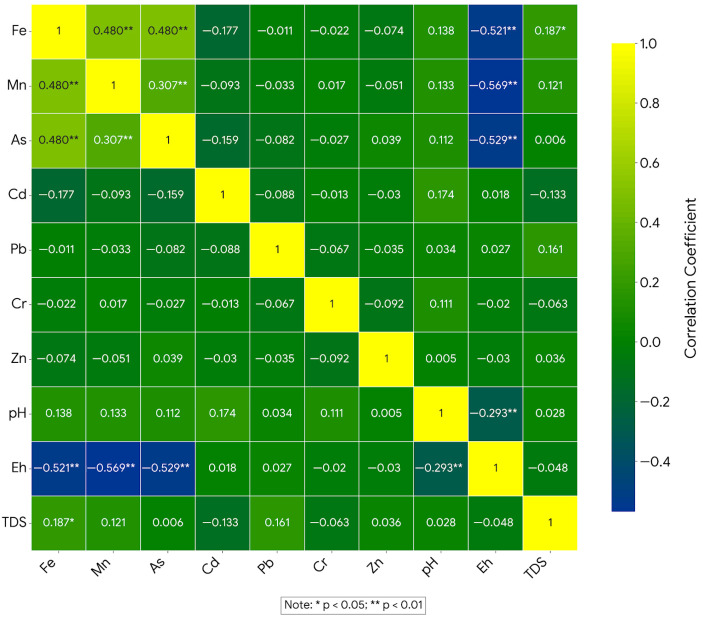
Correlation heatmap of physicochemical parameters and dissolved metals and metalloids.

**Figure 7:**
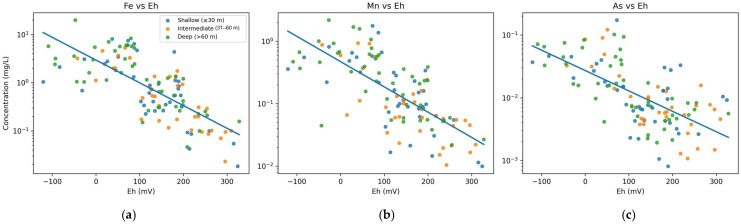
Relationships between redox potential (Eh) and dissolved-metal concentrations: (**a**) iron (Fe); (**b**) manganese (Mn); and (**c**) arsenic (As). The blue line in each panel is the ordinary least-squares (OLS) linear regression of native Eh values vs. log-transformed concentrations.

Pearson correlation analysis indicated a moderate Fe–Mn–As association and an inverse relationship between these metals and metalloids and Eh (Figure 6). Fe was positively correlated with Mn (r = 0.480, *p* < 0.01) and As (r = 0.480, *p* < 0.01), and Mn was positively correlated with As (r = 0.307, *p* < 0.01). All three variables were negatively correlated with Eh (Fe: r = −0.521, Mn: r = −0.569, As: r = −0.529; all *p* < 0.01), as shown in Figure 7. Together with the depth pattern and PCA results, these relationships are consistent with a redox-sensitive co-enrichment pattern, but they do not by themselves prove a unique mechanism.

By contrast, Cd, Pb, Cr, and Zn showed weak or statistically non-significant correlations with Fe, Mn, As, and Eh. Fe showed only a weak positive correlation with TDS (r = 0.187, *p* < 0.05), while Mn and As were not significantly related to TDS. The correlation structure therefore supports one dominant redox-associated association (Fe–Mn–As–Eh) together with weaker secondary patterns for the other metals and metalloids.

This contrast is useful because it reduces the likelihood that all trace metals in the dataset are being controlled by the same hydrogeochemical process. Instead, the correlation structure points to at least two overlapping patterns: a dominant redox-sensitive assemblage involving Fe, Mn, and As (see Figure 7), and a weaker secondary assemblage for the remaining metals. In Figure 7, the blue line in each panel represents the ordinary least-squares (OLS) linear regression fitted to all 120 data points for the respective Eh–metal pair. The regression line was constructed by minimizing the sum of squared vertical residuals between the observed metal concentrations (log-transformed) and the predicted values as a function of Eh. The negative slope of each line reflects the inverse Pearson correlations reported above (Fe: r = −0.521; Mn: r = −0.569; As: r = −0.529; all *p* < 0.01). These trend lines are included to visualize the direction and strength of the linear association rather than to imply a predictive model; scatter around the regression line reflects the natural geochemical heterogeneity of the alluvial aquifer system. Such separation strengthens the interpretation that arsenic behavior in this basin is strongly correlated to redox evolution rather than to generalized total dissolved solids enrichment.

### 3.5. Principal Component Analysis

**Figure 8:**
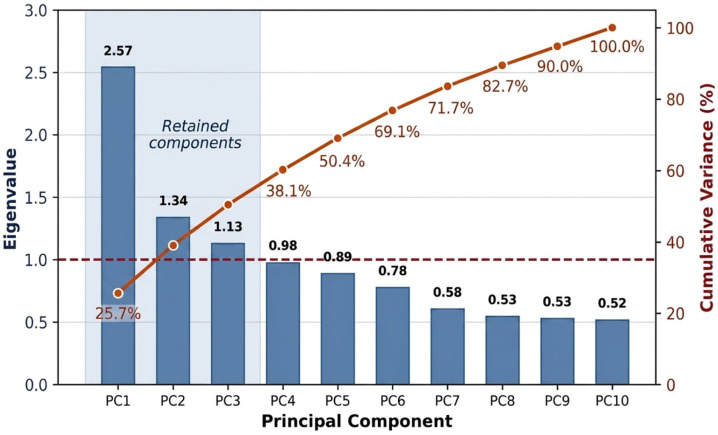
Scree plot showing eigenvalues (bars) and cumulative explained variance (line) for the ten principal components extracted from z-score-standardized hydrochemical data (*n* = 120). The dashed red line indicates the Kaiser criterion (eigenvalue = 1.0). Three components (PC1–PC3) exceeded this threshold and were retained for varimax rotation.

The first three rotated principal components accounted for 50.4% of the total standardized variance (Table 4 and Table 5). The eigenvalues of the retained components were 2.57 (PC1), 1.34 (PC2), and 1.13 (PC3), all exceeding the Kaiser criterion threshold of 1.0 (Table 5). The fourth component had an eigenvalue of 0.98 and was therefore not retained. The scree plot (Figure 8) shows a clear inflection between PC3 and PC4, confirming that three components represent the optimal balance between variance explained and interpretability. PC1 represented a redox-associated groundwater chemistry gradient, with strong positive loadings for Fe (0.795), Mn (0.736), and As (0.743) and a strong negative loading for Eh (−0.838). PC1 therefore captures the dominant hydrogeochemical pattern in the dataset and is consistent with redox-sensitive enrichment in deeper, more reducing groundwater.

PC2 was characterized mainly by pH (0.723), Cd (0.639), and Cr (0.403), suggesting a weaker secondary gradient that may reflect mixed geochemical controls rather than a single definitive source. PC3 was dominated by Pb (0.769) and TDS (0.704), indicating another distinct but weaker component. Taken together, the PCA results support separation between the main Fe-Mn-As redox-associated pattern and other trace-metal behaviors, but they do not by themselves prove specific lithogenic or anthropogenic source pathways.

**Figure 9:**
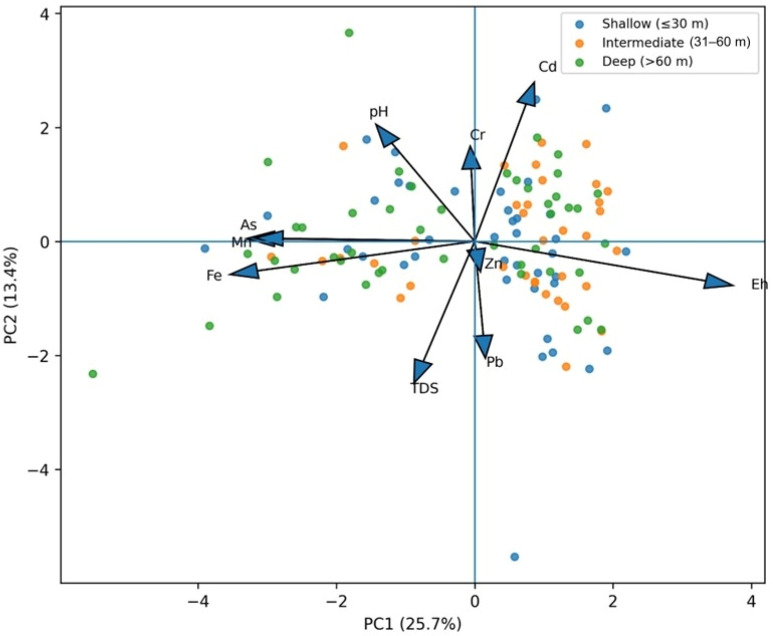
PCA scores and loading biplot.

Figure 9 shows the PCA scores and loading biplot for PC1 versus PC2. The retained components therefore provide a compact summary of basin hydrochemistry: PC1 captures the principal redox gradient, PC2 summarizes a secondary pH–metal relationship, and PC3 represents residual variability associated mainly with Pb and TDS. It is important to note that the cumulative explained variance of 50.4% is moderate rather than exhaustive. This level is, however, consistent with exploratory PCA applications in alluvial groundwater studies where heterogeneous sediments, mixed well types, and spatially variable recharge introduce variance that cannot be captured by a small number of linear components. For context, hydrochemical PCA studies in similar alluvial settings commonly report cumulative explained variances of 45–65% for the first three to four components [33]. The moderate variance explained does not diminish the interpretive value of the retained components; rather, it indicates that the PCA should be used as a pattern-recognition tool to identify dominant covariance structures rather than as an exhaustive source-apportionment model. The PCA results are therefore most valuable when interpreted alongside the correlation matrix and depth patterns rather than in isolation.

### 3.6. Screening-Level Human Health Risk

**Figure 10:**
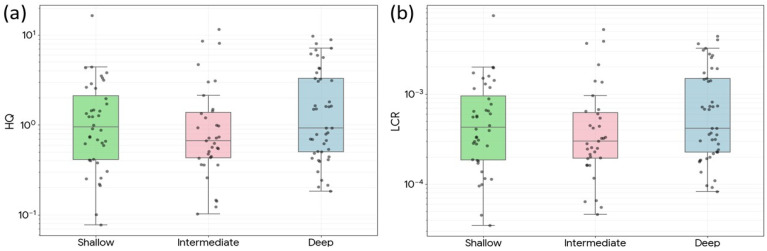
Screening-level arsenic risk plot for adults in different groundwater depth (**a**) HQ (**b**) LCR.

The screening-level ingestion assessment identified arsenic as the dominant toxicological driver in the dataset. Based on the mean arsenic concentration (20.7 μg/L), the estimated hazard quotient was 1.97 for adults and 4.60 for children, exceeding the acceptable threshold of 1.0 in both receptor groups. Mean HQ values for Mn, Cd, and Zn were substantially lower: 0.063, 0.078, and 0.006 for adults and 0.148, 0.182, and 0.015 for children, respectively (Table 6 and Figure 10). The cumulative hazard index for the four-metal screening set (As, Mn, Cd, and Zn) was 2.12 for adults and 4.95 for children.

Lifetime carcinogenic risk, estimated only for inorganic arsenic using the adult resident screening scenario, was 8.87 × 10^−4^ based on the mean arsenic concentration. Using the 90th-percentile arsenic concentration (50.3 μg/L), the estimated lifetime cancer risk increased to 2.15 × 10^–3^, indicating substantially higher concern in the more contaminated wells. Lead was not included in the cumulative HQ because a standard oral RfD is not available in EPA IRIS for routine HQ calculation, but the 20.0% Pb exceedance rate indicates that lead should remain part of the monitoring program.

A well-specific perspective further underscores the importance of arsenic as the primary risk driver. Using the same screening assumptions, 52 of the 120 wells yielded adult arsenic HQ values greater than 1, while 91 wells yielded child arsenic HQ values greater than 1. For cancer risk, 109 wells exceeded 1 × 10^–4^ and 31 wells exceeded 1 × 10^–3^ under the adult resident screening scenario, emphasizing that the basin-wide concern is not restricted to a few isolated outliers.

By contrast, non-carcinogenic risk from the other evaluated metals and metalloids remained comparatively limited at the basin mean, although one well produced a child manganese HQ slightly above 1. This pattern supports the decision to focus the main risk narrative on arsenic while still retaining manganese as an important co-occurring drinking-water concern that may become locally relevant on a well-by-well basis.

## 4. Discussion

### 4.1. Redox-Associated Enrichment of Fe, Mn, and As

The combined depth-profile, correlation, and PCA results support the interpretation that Fe, Mn, and As enrichment in the Chiang Mai Basin is associated with increasingly reducing groundwater conditions. The clearest evidence is the strong inverse relationship between these metals and metalloid and Eh together with the dominant PC1 loadings. This pattern is consistent with the behavior expected in alluvial aquifers where Fe- and Mn-bearing oxide phases become unstable under reducing conditions and release associated trace elements, including arsenic, into groundwater [8,10].

The present dataset does not include dissolved oxygen, sulfate, nitrate, dissolved organic carbon, Fe(II)/Fe(total), or arsenic speciation. The proposed mechanism should therefore be described as evidence-consistent rather than conclusively demonstrated. The observed pattern supports redox-sensitive co-enrichment, but the exact mineralogical and biogeochemical pathways remain to be confirmed in future work.

Even with those limitations, the observed pattern is consistent with the broader literature on alluvial aquifers, where arsenic enrichment commonly develops where redox conditions favor the transformation or dissolution of Fe and Mn oxide phases and the release of previously sorbed or co-precipitated arsenic. Recent field and modeling studies have shown that the exact pathway may vary from site to site, but the repeated association between arsenic occurrence and redox-sensitive iron cycling remains a robust regional theme in sedimentary basins [48,49].

### 4.2. Interpretation of Secondary Metal Patterns

The behaviors of Cd, Pb, Cr, and Zn were distinct from the dominant Fe–Mn–As–Eh pattern. Their generally weak correlations with Eh and with the Fe–Mn–As assemblage indicate that they are not primarily controlled by the same redox-sensitive mechanism. PCA further separated these metals and metalloids into weaker secondary components, with Cd and Cr associated with the pH-related component and Pb associated more strongly with the TDS-dominated component.

These secondary patterns may reflect a combination of aquifer lithology, local mineral weathering, groundwater–sediment interaction and possibly localized anthropogenic influences. However, the results do not justify definitive source apportionment. Statements that these metals and metalloid are proven to be lithogenic or confirmed to derive from a single process should therefore be avoided unless supported by mineralogical, isotopic, or spatial source-tracing evidence.

A practical implication of this finding is that management responses should not assume identical mitigation strategies for all metals and metalloid. The redox-sensitive Fe-Mn-As group may respond to depth-targeted monitoring and treatment approaches that account for co-occurrence, whereas sporadic Cd, Pb, or Cr detections may require source tracing, repeat sampling, or well-construction review to determine whether the driver is geological, anthropogenic, or analytical/transient.

### 4.3. Interpreting CF and PLI in a Groundwater Context

The revised CF and PLI calculations indicate strong departures from the selected global groundwater background values, especially for As and Cd. These indices are useful for summarizing the degree to which the basin dataset differs from a chosen baseline and for comparing depth groups within the same basin. In this study, the highest PLI in deep wells is consistent with the broader evidence for chemically evolved, more problematic groundwater at depth.

Nevertheless, CF and PLI should be interpreted cautiously in groundwater studies. Unlike regulatory thresholds, these indices are highly sensitive to the choice of background concentration. When low global baseline values are used, metals such as As and Cd can generate very high CF values even when many wells remain below drinking-water limits. Accordingly, CF and PLI are best presented as comparative screening tools rather than as stand-alone evidence of severe contamination in a management or regulatory sense.

The revised interpretation is therefore deliberately restrained. High CF or PLI values do not, by themselves, quantify health burden, regulatory non-compliance severity, or source identity. Their main contribution in this manuscript is comparative: they show that As and Cd contribute strongly to background-relative deviation, and that the deep-well group is the most enriched on a composite basis. Those patterns can guide follow-up mapping and monitoring even if the precise numerical magnitude of the indices is baseline dependent.

### 4.4. Screening-Level Human Health Implications

The screening-level risk assessment indicates that arsenic is the principal health concern in the Chiang Mai Basin groundwater dataset. The mean-concentration HQ values for arsenic exceeded 1.0 for both adults and children, and the lifetime arsenic cancer risk exceeded the commonly used screening benchmark of 1 × 10^−4^. The higher HQ for children reflects greater body-weight-normalized intake and is consistent with their higher vulnerability to chronic toxic exposure.

This revised risk interpretation is intentionally conservative but more methodologically consistent than the previous draft. In particular, carcinogenic risk is now presented as a lifetime arsenic risk rather than as separate adult and child cancer risks, Pb is not forced into an HQ framework that is not supported by EPA IRIS, and total Cr is not assigned a cancer interpretation without speciation. Iron is discussed primarily as an acceptability/operational constituent and redox-sensitive indicator rather than as a principal toxicological driver, whereas cadmium is retained in the non-carcinogenic screening framework but not emphasized in the main carcinogenic narrative. These changes align the health-risk section more closely with a defensible screening-level framework.

Recent groundwater risk studies likewise emphasize that screening-level health metrics are most useful when they are paired with transparent assumptions, percentile-based context, and explicit acknowledgment of uncertainty. This is particularly important for alluvial aquifers, where concentrations may vary with season, pumping intensity, and local depositional heterogeneity. This study therefore interprets the calculated HQ, HI, and CR values as decision-support indicators rather than as precise predictions of disease burden [24,25].

The hydrogeological contamination patterns observed in the Chiang Mai Basin align with established global and regional trends. As detailed in Table 7, the contamination of metals and metalloids can be observed in various types of hydrogeologic setting. The Chiang Mai Basin aligns more closely with the geogenic models prevalent across South and Southeast Asia. The transition from shallow unconfined aquifers to deeper confined units similar to the systems described in the Western Lampang Basin and the Lower Sakarya River Basin showed that the deeper groundwater tends to increasing in concentration. In these contexts, the application of risk indices remains a standard for quantifying health impacts; for instance, studies in the Western Lampang Basin and global geogenic assessments report Hazard Quotients (HQ) > 1 and Carcinogenic Risk (CR) values exceeding 10^−4^. These metrics underscore a shared regional burden of high health risks including various cancers, systemic diseases, and neurological disorders associated with groundwater development in both shallow and deep aquifer systems. Table 7. Comparison of hydrogeologic characteristics, dissolved-metal concentrations, contamination sources, and human health risks between the present study and selected groundwater systems worldwide.

### 4.5. Monitoring and Treatment Implications

The results suggest three practical priorities. First, deeper wells should be prioritized for routine monitoring because they show the highest PLI and the strongest Fe–Mn enrichment together with higher mean arsenic concentrations. Second, routine screening should explicitly include As, Mn, Pb, Cd, and Fe, with Fe retained because it is operationally important and can serve as a field indicator of redox-sensitive groundwater evolution. Third, treatment strategies should be selected to address the co-occurring Fe–Mn–As assemblage rather than arsenic in isolation.

Oxidation–coagulation–filtration, adsorption media such as granular ferric hydroxide, and other arsenic-focused treatment systems remain appropriate candidate technologies for community and household application in the basin [2]. Because Pb and Cd occur less coherently than the main redox-sensitive assemblage, targeted verification at the well scale remains important before selecting treatment or drilling controls. Future work should add arsenic and chromium speciation, broader redox indicators, and spatial analysis to better resolve both mechanisms and management priorities.

The treatment discussion was also expanded to reflect recent review literature, which continues to identify adsorption, coagulation-filtration, ion exchange, and membrane processes as the principal treatment families for arsenic-contaminated groundwater. Among these, iron-based media remain especially relevant where arsenic co-occurs with iron and manganese because pretreatment and sequential oxidation-adsorption configurations can often be adapted to mixed contaminant waters. However, technology selection should still be guided by raw-water chemistry, maintenance capacity, waste management, and the scale of implementation [24,50].

## 5. Conclusions

This manuscript evaluates the distribution, hydrogeochemical controls, contamination status, and screening-level health risks of dissolved metals and metalloids in groundwater of the Chiang Mai Basin, Northern Thailand. By integrating depth-based analysis, multivariate statistics, contamination indices, and risk assessment, the study provides a basin-scale framework for interpreting groundwater metal occurrence and its management implications as follows:

1. The Chiang Mai Basin groundwater system is characterized by a redox-sensitive Fe–Mn-As assemblage. Fe, Mn, and As were positively correlated with one another and negatively correlated with Eh, while Fe and Mn increased significantly with depth.
2. Arsenic is the principal screening-level toxicological concern. Mean arsenic hazard quotients exceeded 1.0 for both adults and children, and the estimated lifetime cancer risk was above the commonly applied screening benchmark.
3. Manganese is also a widespread groundwater-quality concern, with 65.0% of samples exceeding the WHO provisional guideline value of 0.08 mg/L. Iron frequently exceeded the 0.3 mg/L acceptability threshold, indicating mainly operational and aesthetic rather than health-based concerns.
4. CF and PLI values indicate substantial deviation from selected global groundwater background concentrations, particularly for As and Cd, with the greatest composite contamination observed in deep wells. These indices are best interpreted as comparative screening tools rather than direct regulatory measures.
5. The results support priority monitoring of deeper wells, routine screening for As, Mn, Pb, and Cd, and treatment strategies designed for the co-occurring Fe-Mn-As assemblage. Future work should address seasonal variability, arsenic and chromium speciation, additional redox indicators, and spatially explicit risk assessment.

Overall, the Chiang Mai Basin represents a hydrogeochemically heterogeneous alluvial groundwater system in which redox evolution, rather than simple mineralization, is the dominant control on the principal toxicological signal. This study demonstrates the value of integrating basin-scale screening, depth-stratified hydrogeochemical interpretation, and health-risk assessment to support groundwater management.

## Figures and Tables

**Table 1:** Descriptive statistics of hydrochemical parameters and dissolved metals and metalloids by groundwater depth group. Values are shown as mean ± SD (min–max), together with the applicable drinking-water guideline or threshold and the percentage of samples exceeding that benchmark.

Parameter	Unit	Shallow (*n* = 40)	Intermediate (*n* = 35)	Deep (*n* = 45)	All Wells (*n* = 120)	WHO Std.	% Exceed.
pH	–	7.19 ± 0.45 (6.48–8.22)	7.17 ± 0.47 (6.00–8.12)	7.26 ± 0.57 (6.12–8.80)	7.21 ± 0.50 (6.00–8.80)	6.5–8.5	5.8
Eh	mV	123.8 ± 95.4 (−120.7 to +324.1)	161.8 ± 91.9 (−42.3 to +309.2)	98.3 ± 104.1 (−108.8 to +327.7)	125.3 ± 100.4 (−120.7 to +327.7)	–	–
TDS	mg/L	480.9 ± 169.7 (239.3–992.0)	474.5 ± 141.4 (231.8–803.0)	495.7 ± 185.5 (257.1–1111.5)	484.6 ± 167.2 (231.8–1111.5)	500	40.0
Fe	mg/L	1.684 ± 2.160 (0.018–8.119)	1.077 ± 1.377 (0.023–5.305)	2.614 ± 3.498 (0.046–20)	1.856 ± 2.646 (0.018–20)	0.3 *	72.5
Mn	mg/L	0.293 ± 0.368 (0.010–1.770)	0.180 ± 0.265 (0.010–0.931)	0.427 ± 0.474 (0.022–2.186)	0.311 ± 0.397 (0.010–2.186)	0.08	65.0
As	µg/L	18.6 ± 28.2 (0.8–173.0)	17.7 ± 27.6 (1.1–121.6)	25.0 ± 27.7 (1.9–101.9)	20.7 ± 27.8 (0.8–173.0)	10.0	45.8
Cd	µg/L	1.3 ± 1.1 (0.1–4.8)	1.3 ± 1.0 (0.1–3.4)	1.5 ± 1.0 (0.4–5.6)	1.4 ± 1.1 (0.1–5.6)	3.0	11.7
Pb	µg/L	7.9 ± 9.5 (0.8–54.5)	5.9 ± 3.5 (0.9–16.1)	6.2 ± 4.3 (0.7–18.2)	6.7 ± 6.4 (0.7–54.5)	10.0	20.0
Cr	µg/L	7.0 ± 6.1 (0.9–31.3)	7.1 ± 7.6 (0.6–41.4)	7.5 ± 6.2 (0.5–27.6)	7.2 ± 6.6 (0.5–41.4)	50.0	0.0
Zn	µg/L	70.6 ± 70.0 (9.6–344.5)	57.8 ± 41.3 (12.8–170.0)	69.8 ± 54.5 (12.0–228.8)	66.5 ± 56.8 (9.6–344.5)	–	–
Hg	µg/L	0.3 ± 0.2 (0.0–1.1)	0.5 ± 0.4 (0.1–2.1)	0.4 ± 0.5 (0.0–2.6)	0.4 ± 0.4 (0.0–2.6)	6.0	0.0
Se	µg/L	3.2 ± 2.4 (0.6–9.8)	3.7 ± 3.3 (0.6–12.2)	3.5 ± 3.1 (0.6–13.1)	3.5 ± 2.9 (0.6–13.1)	40.0	0.0

* Iron is reported against the 0.3 mg/L acceptability/operational threshold rather than a health-based WHO guideline value. – Eh and Zinc are not establishing WHO guideline value because they are not of health concern at levels found in drinking-water.

**Table 2:** Kruskal–Wallis test results for hydrochemical parameters and dissolved metals and metalloids among the three groundwater depth groups.

Parameter	H	*p*-Value	Significance
Fe	7.60	0.0224	*
Mn	10.75	0.0046	**
Zn	0.55	0.7611	ns
Cd	3.98	0.1366	ns
Hg	5.40	0.0673	ns
Cr	0.87	0.6463	ns
As	3.15	0.2067	ns
Se	0.09	0.9546	ns
Pb	0.10	0.9525	ns
Eh	7.09	0.0289	*
pH	0.20	0.9043	ns
TDS	0.03	0.9854	ns

Significance notation: * *p* < 0.05, ** *p* < 0.01, ns = not significant.

**Table 3:** Contamination factors (CF) and pollution load index (PLI) for the evaluated metals using selected global groundwater background values. CF and PLI were calculated for the full-dataset mean concentration (overall) and average of the three depth-group concentrations (shallow, intermediate, and deep).

Metal	Concentration (μg/L)		CF				
	Background	Overall Mean	Overall CF	Class	Shallow CF	Intermediate CF	Deep CF
Fe	200.0	1856.00	9.28	Very High (≥6)	8.42	5.38	13.07
Mn	50.0	311.00	6.21	Very High (≥6)	5.87	3.60	8.54
As	1.0	20.70	20.71	Very High (≥6)	18.56	17.66	24.99
Cd	0.1	1.37	13.65	Very High (≥6)	12.66	13.10	14.96
Pb	1.0	6.68	6.68	Very High (≥6)	7.93	5.93	6.15
Cr	1.0	7.20	7.20	Very High (≥6)	6.95	7.14	7.46
Zn	10.0	66.50	6.65	Very High (≥6)	7.06	5.78	6.98
**PLI**	-	-	9.11	-	8.93	7.29	10.42

Note: CF and PLI are interpreted as background-relative screening metrics; they are sensitive to the choice of baseline values.

**Table 4:** Eigenvalues and explained variance for all principal components. Components retained under the Kaiser criterion (eigenvalue > 1.0) are shown in bold.

Component	Eigenvalue	Variance (%)	Cumulative (%)
**PC1**	**2.570**	**25.7**	**25.7**
**PC2**	**1.340**	**13.4**	**39.1**
**PC3**	**1.130**	**11.3**	**50.4**
PC4	0.980	9.8	60.2
PC5	0.890	8.9	69.1
PC6–PC10	0.78–0.52	5.2–7.8	100.0

Notes: Bold rows indicate retained components (eigenvalue > 1.0, Kaiser criterion). The eigenvalue of PC4 (0.98) falls just below the threshold, and the scree plot inflection confirms the three-component solution.

**Table 5:** Varimax-rotated PCA loadings for the three retained components.

Variable	PC1	PC2	PC3
Fe	0.795	−0.062	0.141
Mn	0.736	0.074	0.079
As	0.743	−0.142	−0.166
Cd	−0.215	0.639	−0.234
Pb	−0.106	0.066	0.769
Cr	−0.000	0.403	−0.115
Zn	0.011	−0.287	−0.141
pH	0.269	0.723	0.120
Eh	−0.838	−0.193	0.032
TDS	0.146	−0.127	0.704
Explained variance (%)	25.7	13.4	11.3
Cumulative variance (%)	25.7	39.1	50.4

**Table 6:** Screening-level ingestion risk metrics based on mean dissolved concentrations in the 120-well dataset. Reported ADD, HQ, and HI values are mean-concentration screening estimates for adults and children under the assumed exposure parameters.

Metal	Mean Concentration (mg/L)	RfD (mg/kg/Day)	ADD		HQ		Interpretation
			Adult	Child	Adult	Child	
As	0.0207	3.0 × 10^–4^	5.92 × 10^–4^	1.38 × 10^–3^	1.97	4.60	Primary concern
Mn	0.3105	1.4 × 10^–1^	8.87 × 10^–3^	2.07 × 10^–2^	0.063	0.148	Low relative to As
Cd	0.0014	5.0 × 10^–4^	3.90 × 10^–5^	9.10 × 10^–5^	0.078	0.182	Low relative to As
Zn	0.0665	3.0 × 10^–1^	1.90 × 10^–3^	4.44 × 10^–3^	0.006	0.015	Low relative to As
HI total	-	-	-	-	2.12	4.95	Cumulative hazard index
As lifetime CR	0.0207	1.5 (CSF)	-	-	8.87 × 10^–4^	-	Mean-concentration cancer risk
As lifetime CR (P90)	0.0503	1.5 (CSF)	-	-	2.15 × 10^−3^	-	Higher-risk wells

Notes: Pb was not included in the cumulative HQ because EPA IRIS does not provide a standard oral RfD suitable for routine HQ derivation for inorganic lead. Total Cr was not included in cancer-risk calculations because chromium speciation was unavailable.

**Table 7:** Comparison of hydrogeologic settings, dissolved-metal concentrations, health risk indices, and contamination metrics between the Chiang Mai Basin (this study) and selected alluvial and sedimentary groundwater systems in Asia and globally. Emphasis is placed on arsenic as the principal toxicological constituent and on depth-dependent enrichment patterns in geogenically influenced aquifer systems.

Location	Hydrogeologic Characteristics	Dissolved-Metal Concentrations	Human Health Risk	PLI/Related Indices	References
Chiang Mai Basin	An intermontane basin with a central floodplain covered by unconsolidated sediments	As: 18.56 μg/L (shallow), 17.66 μg/L (intermediate); 24.99 μg/L (deep);	High: Carcinogenic risk of 8.87 × 10^–4^	Hazard Quotient (HQ) > 1, indicating high potential adverse health risks.	This work.
Western Lampang Basin, Northern Thailand	An intermontane basin with a central floodplain covered by unconsolidated sediments and semi-consolidated basements.	As:<2.8–35 μg/L (shallow), <2.8–480 μg/L (deep); Fe: avg 0.6 mg/L (shallow), up to 68 mg/L (deep);	High: Carcinogenic risk of 2.78 × 10^−3^	Hazard Quotient (HQ) > 1, indicating high potential adverse health risks.	Santha et al., 2022 [7]
Indian Peninsula	unconsolidated sedimentary aquifers	As: Typically > 10 μg/L (WHO limit).	High: Skin, lung, kidney, and bladder cancer; coronary heart disease; hyperkeratosis; arsenicosis.	-	Shaji et al., 2021 [1]
Palar River Basin, Tamil Nadu, India	Aquifer specific details are not heavily defined, but it is an industrially impacted river basin where heavy metals have severely degraded.	Significant contamination of Cr, Co, Cu, Cd, Fe, and moderate contamination of Mn, Ni, Zn (specific concentrations not listed).	Extremely High: Mental illness, stomach and skin cancer, liver failure, and kidney damage.	Health Risk Assessment Index (HRAI) = 9028.6; also utilized Heavy Metal Index, Contamination Factor, and Igeo Index.	Dange et al., 2024 [12]
Lower Sakarya River Basin, Turkey	A fault-bounded alluvial plain with a shallow unconfined/semi-confined aquifer.	As: up to 373 μg/L (deep aquifer); Mn: up to 1230 μg/L (deep aquifer); Ni: up to 56.9 μg/L (shallow).	High: Elevated arsenic and manganese levels pose a major global public health threat via drinking water.	33–41% (As) and 44% (Mn) of deep aquifer samples exceeded the Maximum Contaminant Levels (MCLs).	Talay & Yolcubal, 2025 [49]
South and Southeast Asia	Alluvial and deltaic sediment aquifers, including the Ganges-Brahmaputra, Mekong, and Red River floodplains.	As: High levels in shallow alluvial aquifers.	High: Severe carcinogen exposure for tens of millions via daily drinking water and irrigation.	-	Fendorf et al., 2010 [9]

## Data Availability

The data presented in this study are available on request from the corresponding author.

## Supplementary Materials

The following supporting information can be downloaded at https://www.mdpi.com/article/10.3390/toxics14050390/s1: Table S1: Sampling well information; physicochemical and metal contents for all samples.
